# Combined targeting of co-stimulatory (OX40) and co-inhibitory (CTLA-4) pathways elicits potent effector T cells capable of driving robust anti-tumor immunity

**DOI:** 10.1186/2051-1426-1-S1-P87

**Published:** 2013-11-07

**Authors:** William L Redmond, Stefanie N Linch, Melissa J Kasiewicz

**Affiliations:** 1 Cancer Research, Earle A. Chiles Research Institute, Portland, OR, USA

## 

Ligation of the TNF receptor family co-stimulatory molecule OX40 (CD134) with an agonist anti-OX40 mAb enhances anti-tumor immunity by augmenting T cell differentiation as well as turning off the suppressive activity of FoxP3+CD4+ regulatory T cells (Treg). In addition, antibody-mediated blockade of the checkpoint inhibitor, CTLA-4, releases the “brakes” on T cells to augment tumor immunotherapy. However, monotherapy with these agents can have limited therapeutic benefit particularly against poorly immunogenic murine tumors. Therefore, we examined whether the combination of agonist anti-OX40 therapy in the presence of CTLA-4 blockade would enhance tumor immunotherapy. Combined anti-OX40/anti-CTLA-4 immunotherapy significantly enhanced tumor regression and the survival of tumor-bearing hosts in a CD4 and CD8 T cell-dependent manner. Mechanistic studies revealed that combination immunotherapy directed the expansion of effector T-bethigh/Eomeshigh granzyme B+ CD8 T cells. Dual immunotherapy also induced among distinct populations of Th1 (IL-2, IFN-γ) and, surprisingly, Th2 (IL-4, IL-5, and IL-13) CD4 T cells exhibiting increased T-bet and Gata-3 expression. Furthermore, IL-4 blockade inhibited the Th2 response, while maintaining Th1 CD4 and effector CD8 T cells that enhanced tumor-free survival. These data demonstrate that refining the global T cell response during combination immunotherapy can further enhance the therapeutic efficacy of these agents.

